# Raw meat-based diets for dogs: survey of owners’ motivations, attitudes and practices

**DOI:** 10.1186/s12917-019-1824-x

**Published:** 2019-03-04

**Authors:** Giada Morelli, Sofia Bastianello, Paolo Catellani, Rebecca Ricci

**Affiliations:** Department of Animal Medicine, Production and Health, University of Padua, Viale dell’Università 16, 35020 Legnaro, PD Italy

**Keywords:** Dog, Diet, Nutrition, Owner, Raw, Survey

## Abstract

**Background:**

The popularity of raw meat-based diets (RMBDs) for pets has been increasing in recent years even if the reputed health benefits are mainly anecdotal. A web-based survey was developed to better understand the motivations and habits of owners who decided to feed their dogs RMBDs.

**Results:**

The questionnaire was completed by 218 dog owners, 62 of whom were living with people whose immune system was impaired or weakened. Internet was the preferred source of information for more than half of the respondents, and feeding dogs a more natural and healthier diet was the main reason behind owners’ interest in RMBDs. About 80% of the participants completely abandoned commercial pet food and showed marked distrust especially towards the lack of clarity on the ingredients used (*n* = 169). The vast majority of owners interviewed (94%) believed RMBDs to be absolutely safe for dogs, and shinier coat, muscle mass gain, and cleaner teeth were the principal improvements seen on their pets. Controlling the composition and quality of the ingredients provided to their animals was the main advantage of RMBDs for 57% of the owners, while the main disadvantages were related to the purchase of some components (38%) and the time required (22%) for the preparation of the diet. Only 8% of the respondents relied on veterinarians for RMBD formulation, and a wide variety of feeding regimens and combinations of ingredients was observed.

**Conclusions:**

As revealed by this study, most owners are unaware of the risks posed by the feeding of RMBDs for both animal and human health, and they often rely on questionable sources for advice on pet nutrition. Owners see RMBDs as a more natural and healthier alternative to commercial pet food even if the actual benefits remain unproven. Consulting veterinarians for proper information and board-certified nutritionists for the formulation of complete and balanced RMBDs should be promoted.

**Electronic supplementary material:**

The online version of this article (10.1186/s12917-019-1824-x) contains supplementary material, which is available to authorized users.

## Background

Feeding raw meat-based diets (RMBDs) to dogs and cats has become a widespread practice in recent years [1, 2]. Many owners have become supporters of diets for pets prepared with fresh uncooked ingredients like meat, offal, and bones as an alternative to commercial dry or canned pet food, and certain health benefits are frequently reported by owners. Despite the increasing popularity of RMBDs, few studies have been conducted on the health effects on pets, and the real advantages have not yet been clearly proven [3]. On the contrary, some undesired consequences of feeding RMBDs to dogs and cats have been well documented. Firstly, the risk of pets consuming incomplete and unbalanced daily meals is high, given that most recommendations for feeding RMBDs published have not been peer reviewed and are not based on the opinion of individuals with proper nutritional expertise [3]; consequently, the deficiency or excess of certain nutrients could give rise to health problems, especially in young animals. Secondly, as shown in literature, raw meat and internal organs can be easily contaminated during slaughter, processing or transportation, and often, in fact, test positive for pathogenic bacteria, viruses and parasites [2–5]. The spread of zoonotic bacterial pathogens either from contaminated raw meat products or from the feces of pets fed RMBDs has been incontrovertibly demonstrated and therefore poses concrete risks to the health of the people handling raw meat products while preparing the diet or simply living in contact with animals consuming RMBDs [2–5]. Major concerns arise especially for individuals with impaired or weakened immune systems (i.e. children, chronically ill people, elderly people and pregnant women) because such category of individuals is more susceptible to developing foodborne infections [3–5].

The aims of this study were to better determine the motivations and the feeding practices of people who routinely administer RMBDs to their dogs.

## Results

### Survey participants

The survey permitted 218 dog owners to be self-recruited (Table 1). Most respondents were women between 21 to 40 years old (51%, 112/218). Sixty-two households (28%) included one or more individuals whose immune system was either impaired or not yet fully developed, such as children, the chronically ill, the elderly, and pregnant women.

**Table 1:** Demographics of survey respondents (*n* = 218)

	Dog owners, n (%)
Gender	
Male	40 (18)
Female	178 (82)
Age, years old	
< 20	6 (3)
21–40	142 (65)
40–60	70 (32)
> 60	1 (0)
Household	
Single	44 (20)
Couple	105 (48)
Family	60 (28)
Other	9 (4)
People at risk in the household	
Children < 3 years old	12
Children 3–10 years old	29
Elderly > 75 years old	19
Pregnant women	4
Chronically ill	21
None	132

### Canine population

Thirty-nine dogs involved in this survey were mongrel; the remainder belonged to 60 breeds, of which the most amply represented were Labrador and Golden Retriever, Czechoslovakian Wolfdog, German Shepherd, Border Collie, Akita Inu, and Australian Shepherd; all other breeds accounted for less than 3% of the dogs considered. As per the national standard, medium and large size breeds prevailed with 61 and 83 dogs, respectively, while 20 dogs were small size and 15 giant size. Males and females were equally represented, most of which were intact. The mean dog age was 3.8 years old (SD: 13.0) and ranged from 0.1 to 15.0 years old. Most dogs (86%) had an ideal body condition in their owner’s perception. Table 2 provides a summary of dog demographics. The majority of the dogs enrolled (77%) was reported to live inside the house all day, while 17% lived indoors only a few hours a day and 6% lived outdoors all the time.

**Table 2:** Characteristics of dogs enrolled in the study (*n* = 218)

	Dogs, n (%)
Gender	
Male	112 (51)
Female	107 (49)
Neutering status	
Neutered	72 (33)
Not neutered	147 (67)
Age, years old	
< 1	29 (14)
1–2	63 (29)
2–7	93 (44)
7–12	25 (12)
> 12	3 (1)
Weight, kg	
Mean ± SD	27.2 ± 13.5
Median (Range)	27.0 (1.0–78.0)
Body condition (according to owner)	
Underweight	19 (9)
Normal weight	187 (86)
Overweight	12 (5)
Most represented breeds (8 out of 61)	
Mongrel	39 (18)
Golden Retriever	19 (9)
Czechoslovakian Wolfdog	14 (6)
German Shepherd	14 (6)
Labrador Retriever	11 (5)
Border Collie	9 (4)
Akita Inu	8 (4)
Australian Shepherd	7 (3)

### Owner motivation and attitude

Many owners interviewed had chosen diets containing raw products by searching the internet for information (60%, 130/218) or consulting magazines and books (19%, 42/218); for some owners, the feeding of RMBDs was encouraged by breeders (12%, 26/218) or veterinarians (9%, 19/218); 8 owners (4%) gave other answers. The respondents were asked to indicate the main reason they chose to provide RMBDs to their dogs: 26% (58/218) said to respect the dog’s carnivorous nature, 24% (52/218) to improve the pet’s health condition, 21% (46/218) because commercial pet food had caused problems in the past, 19% (41/218) because they did not trust commercial pet food, 6% (13/218) because their dog did not eat commercial pet food, and 4% (8/218) stated another reason. As regards the use of traditional pet food, 79% (173/218) of the owners completely eliminated commercial dry food, while 12% (26/218) still used it when necessary (e.g. when not at home) and 9% (19/218) regularly. Almost every owner (94%, 206/218) had had at least one previous experience with commercial pet food and were asked which features were the most undesirable: 169 respondents (78%) said the scarce information on the ingredients (e.g. origin, quality), 101 (46%) the inclusion of additives, and 38 (17%) the presence of carbohydrates; some owners reported that their dogs were not satisfied because they consumed their meals too fast (13%, 28/218) or did not like their food (7%, 16/218); 21 owners (10%) gave other answers; on the other hand, 66 owners (30%) said they liked preparing food for pets and 36 (17%) preferred giving pets fresh ingredients.

**Fig. 1:**
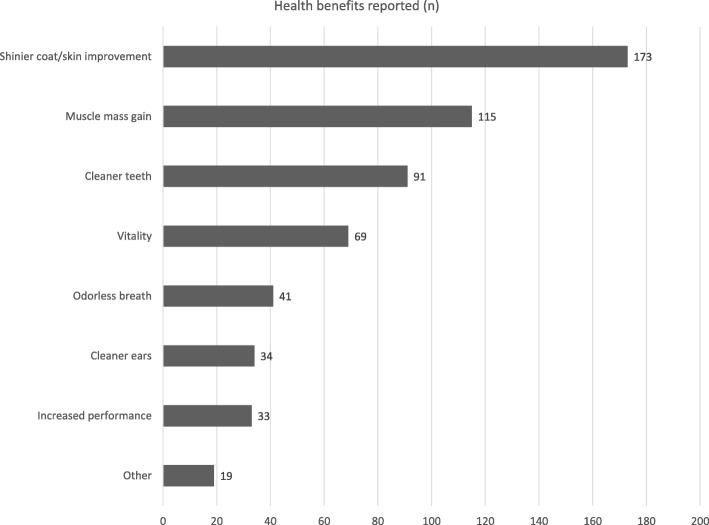
Health benefits of RMBDs reported by dog owners interviewed

Most owners reported health improvements in their pets, including a shinier coat, muscle mass gain, and cleaner teeth (Fig. 1). As regards the dog’s behavior, 51% (112/218) of the owners reported no abnormalities, while the remaining noticed a calmer (35%, 76/218) or livelier (21%, 45/218) attitude; no one reported nervousness or aggression.

**Fig. 2:**
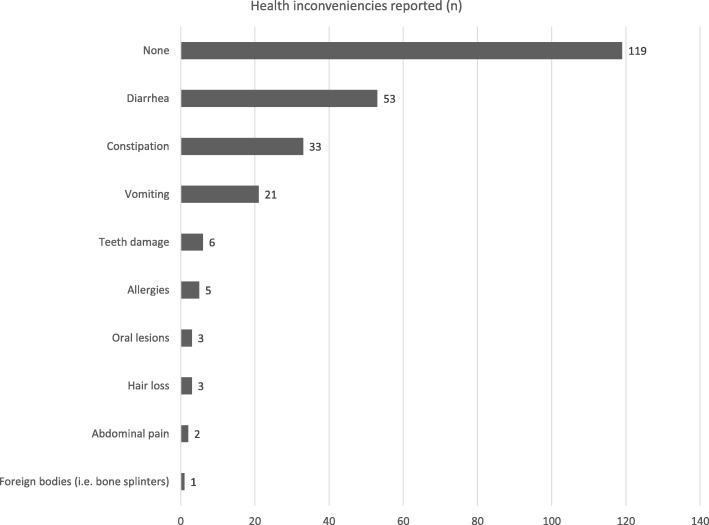
RMBD-related health issues reported by dog owners interviewed

Almost all respondents (94%, 206/218) said they consider RMBDs safe for pets, while only 1% (2/218) acknowledged the possibility of health risks associated with this kind of diet and 5% (11/218) never raised the issue. Additionally, 141 owners (65%) stated that dogs cannot get ill by consuming a RMBD, 38 (17%) that it is possible but no reason for worry, and 10 (5%) had never heard of this possibility; on the contrary, 29 persons (13%) said dogs can get ill by eating RMBD. Fifty-five percent of the owners interviewed (119/218) never noticed side effects after eating RMBDs, while the remaining 45% reported mainly diarrhea, constipation, and vomiting (Fig. 2). Owners were also asked if they would refuse the administration of RMBDs in certain conditions: 8 (4%) said for puppies, 5 (2%) for senior dogs, 5 (2%) for dogs with health problems, 2 for pregnant bitches and 2 for lactating bitches (1% each).

As the main advantage of feeding dogs with a RMBD, 57% of the owners interviewed (125/218) reported the total control over the diet and awareness of its composition, while 23% (51/218) mentioned animal origin proteins as principal component and 11% (23/218) the long time it requires to be eaten by the dog and the animal’s consequent greater satisfaction. Only few owners considered good palatability (3%, 6/218), the absence of carbohydrates (1%, 2/218) or the rawness of the ingredients (1%, 2/218) as the main advantage, and 4% (9/218) gave different answers. As main drawbacks, 38% (83/218) reported having to procure certain ingredients, 22% (48/218) the amount of time required for preparation, 13% (28/218) the cost, 5% (10/218) the risk of bone ingestion, 4% (9/218) the dog’s loss of appetite with some ingredients, and 2% (5/218) diarrhea or vomiting. Further disadvantages were reported under the “other” options (16%, 35/218), among which prevailed the difficulty of feeding the dog a RMBD when the animal is looked after by someone else and the large space occupied by the ingredients stored in the refrigerator.

### Owner practices and RMBD formulation

Most RMBDs were formulated by the owners themselves following other people’s advice available online (33%, 71/218), using nutritional guidelines published in websites or in books (31%, 67/218), or following no rules (19%, 41/218). Only 8% (18/218) turned to the veterinarian and 5% (12/218) to a nutritionist for RMBD formulation, and 1% (3/218) relied on guidelines provided by online raw meat product sellers; 3% (6/218) gave other answers.

**Fig. 3:**
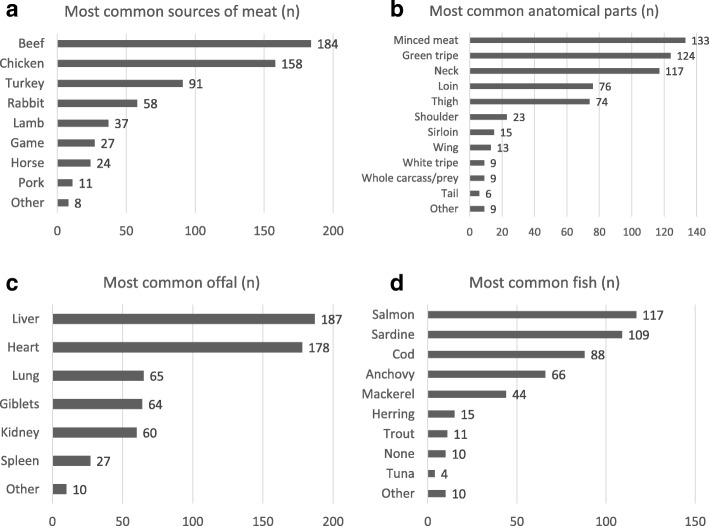
The most common meat (**a**), anatomical parts (**b**), offal (**c**) and fish (**d**) purchased by the RMBD-feeders interviewed

Respondent owners affirmed buying raw meat products at the butcher shop (47%, 102/218), at the supermarket (36%, 78/218), at online shops (28%, 62/218), at the slaughterhouse (17%, 38/218) or at any of these indistinctly (29%, 63/218). Figure 3 shows the most common raw meat products used by the respondents. The most frequently purchased meat types were beef, chicken and turkey, while the most common meat parts were skeletal muscle (i.e. minced meat), green tripe, and chicken neck, back and thigh. Among the offal, liver and heart were the most widely used. Also, 95% (208/218) of the owners regularly used raw fish, among which salmon, sardines and cod prevailed. As regards bones, 41% (90/218) of the owners gave them to their dogs daily, 41% (90/218) once or twice a week, 10% (22/218) rarely, and 8% (17/218) never. Other commonly used animal origin ingredients were eggs (85%, 185/218), yoghurt (59%, 128/218), cheese (34%, 74/218), milk (11%, 24/218), lard (5%, 11/218), butter (3%, 7/218), tallow (2%, 4/218) and other (9%, 20/218). Although carbohydrates were generally not used in the diet (52, 113/218), some reported adding potatoes (23%, 51/218), bread (18%, 39/218), rice (regular, 15%, 33/218; puffed, 12%, 27/218), pasta (6%, 12/218) or other cereals (18%, 39/218). Owners were also asked whether they used vegetables and fruit, and the answers were not homogeneous. Carrots (91%), zucchini (87%), leafy vegetables (75%) squash (58%) and celery (57%) were the vegetables most commonly used, but not on a daily basis (circa once a week) (Table 3). Similarly, the most common fruit were apple (89%), banana (65%) and pear (57%), generally administered as a snack one to four times per month (Table 4).

**Table 3:** Vegetables used by participating dog owners (*n* = 218)

	Never	Rarely (1–3 times/month)	Once a week	Often (2–3 times/week)	Daily
Artichokes	88	10	1	1	0
Beans	83	13	2	1	1
Bell peppers	87	9	3	1	0
Cabbage	60	26	10	2	2
Carrots	9	16	28	30	17
Celery	43	25	18	9	5
Eggplant	92	6	1	1	0
Green beans	64	20	11	5	0
Leafy vegetables	25	30	20	16	9
Peas	69	19	8	3	1
Squash	42	35	13	7	3
Zucchini	13	19	26	29	13

**Table 4:** Fruit used by participating dog owners (*n* = 218)

	Never	Rarely (1–3 times/month)	Once a week	Often (2–3 times/week)	Daily
Apple	11	24	28	26	11
Apricot	61	28	7	3	1
Banana	35	37	17	8	3
Citrus	67	25	5	3	0
Fig	83	11	4	2	0
Melon	59	25	10	4	2
Peach	56	27	12	5	0
Pear	43	25	18	12	2
Pineapple	76	18	4	2	0
Strawberry	78	17	2	2	1
Watermelon	52	32	10	5	1

**Fig. 4:**
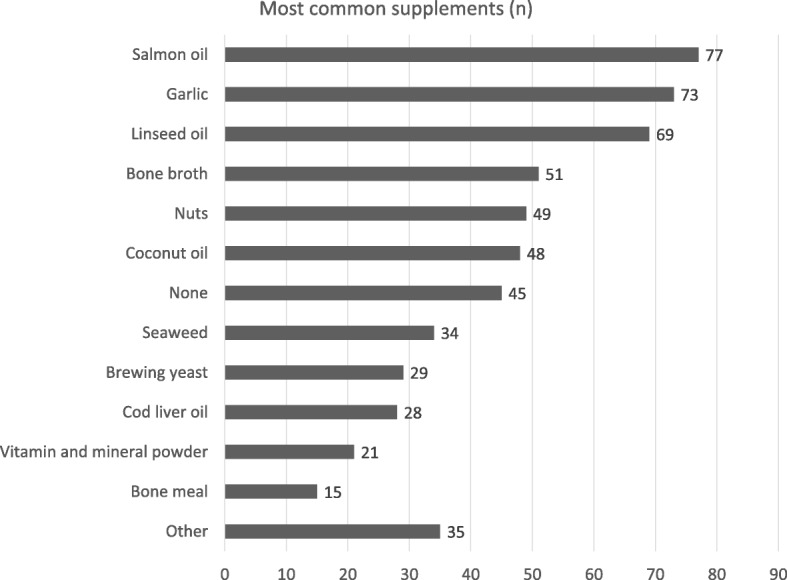
The most common ingredients used as supplements by the RMBD-feeders interviewed

The owners interviewed were found to use many other ingredients as supplements, among which salmon oil, garlic and linseed oil were the most common (Fig. 4); only 21 owners (10%) reported supplementing RMBDs with a vitamin and mineral supplement, and 45 (21%) said they used no other ingredient or supplement than the ones already mentioned in the questionnaire.

Mean daily RMBD preparation time was 10 min for 110 owners (50%), 20 to 30 min for 40 owners (18%), 30 to 60 min for 6 owners (3%) and 60 to 120 min for one owner (1%); 42 owners (19%) stated they were unable to quantify the time spent on RMBD preparation, whereas 19 (9%) simply waited for frozen prepacked RMBDs to defrost.

## Discussion

Despite the growing popularity of RMBDs in recent years, few studies have been conducted to gather information on the motivations and habits of people who choose to feed their pets raw meat. Information collected in this survey shows that dogs fed RMBDs had a common profile: they were mainly intact, young, and belonged to medium and large size breeds. The most relevant demographic data gathered on the owners interviewed was that 28% lived with pregnant women, young children, or older adults and chronically ill people. Such individuals are at greater risk of developing foodborne illness and should therefore minimize the handling of undercooked or raw meat products that are commonly contaminated by a variety of pathogens [2–5]. Also, animals fed raw food diets may be a potential cause of illness in susceptible people due to the environmental shedding of zoonotic pathogenic bacteria and parasites [4, 6]. Interaction with all family members is inevitable, given that the majority of the respondents’ dogs was reported to live inside the house all day; therefore, the feeding of RMBDs should be reconsidered in households with individuals at risk. The consumption of raw meat, in fact, has been strongly discouraged for dogs enrolled in pet therapy programs and interacting with people having compromised immune systems [7]. Bearing in mind that RMBDs may have a negative impact on both pet and owner health, the veterinarian’s role in the nutritional education of the client should not be underestimated.

Most of the respondents affirmed relying on information sources other than veterinarians and, according to other surveys [8–10], an important percentage of owners consult websites to learn how to feed pets nowadays. This is especially true for owners who do not feed commercial pet food owing to their distrust in the veterinarian’s nutritional expertise [8, 11–13]. This could lead to misinformation on the appropriateness of RMBDs, their virtues and safety, because content available in websites, books and magazines may be biased by the author’s opinion and level of scientific background. Promoting the importance of consulting a veterinarian and greater dissemination of the proven health implications of RMBDs in the mass media could help pet owners to make more informed science-based decisions [14]. However, although the majority of veterinarians adopt a critical stance towards the practice of raw feeding, 9% of the respondents reported contacting veterinarians in favor, in demonstration of the fact that the profession is divided – albeit unevenly – on the issue of feeding pets raw meat [1].

The main reasons that respondents switched to a RMBD for their pets are evenly distributed instead: 1) to respect the dog’s ancestral carnivorous nature, 2) to give the dog a healthier diet, 3) to avoid commercial pet food. These three principles were also significantly adopted by RMBD-feeding owners surveyed in previous studies [8, 11, 14]. Providing a diet that mimics the one that wild, non-domesticated dogs ate before their evolution into pets is popular rationale in owners who support RMBDs. In our opinion, RMBDs clearly resemble the wolf’s diet more closely than kibbles, and this meets the dog’s behavioral needs, which are seriously taken into account by owners. From a nutritional point of view, however, it must be borne in mind that the domestication of the dog implied genetic modifications that enabled its metabolism to differ significantly from its ancestor’s: the modern dog’s clearly demonstrated ability to digest starch is a good example [15]. Therefore, although feeding RMBDs for the behavioral aspect sounds reasonable, reducing owners’ preference for this type of diet to merely a way of excluding carbohydrates may be simplistic. Moreover, the choice of feeding a RMBD also implies accepting certain features (e.g. ingredient contamination and bone consumption) that may not be optimal for pet dogs living in a home environment [3]. The second main reason for switching to RMBDs, improving the pet’s health, was also cited by the persons interviewed by Morgan and colleagues [8]. Despite the owners’ firm opinion, however, the reputed health and therapeutic benefits of raw diets are mainly anecdotal and sufficient scientific validation has yet to be made [3]. Lastly, owners feeding noncommercial diets firmly support the nutritional superiority of RMBDs over commercial pet food, about which they generally tend to have more concerns and misgivings [11]. In fact, even if one out of five owners interviewed continued using commercial dry pet food (more or less regularly) due to its convenience, most abandoned it permanently. Whether some owners used wet pet foods or cooked meats along with the RMBD was not investigated. Undeniably, pet food does not always allow clear identification of its ingredients and has been the target of scandals (e.g. melamine inclusion, contamination, fraud) [16]. The scarce information provided on the ingredients used and the inclusion of additives such as preservatives and colorants were, in fact, the most displeasing features of commercial pet food reported in our survey. In this respect, the possibility for owners to feed their dogs meals prepared with unprocessed fresh ingredients is a reasonable point in favor of RMBDs. However, it is difficult to compare the safety and quality of pet food and RMBDs because the latter have a more limited market and lack rigorous quality controls, and their associated illnesses probably go unreported and therefore underrated [3].

The most common health benefits reported here were shinier coat, muscle mass improvement, and cleaner teeth. At present, only one recent study by Marx and colleagues [17] demonstrated that daily supplementation with raw bovine bones (i.e. bovine raw cortical bone from femur diaphysis) reduced the amount of dental calculus up to 70.6% after 12 days in eight beagle dogs; unlike other studies [18–20], teeth fractures were not reported. As already discussed however, the abovementioned benefits remain unproven and future studies are needed to clarify the positive aspects of feeding pets RMBDs. On the other hand, 45% of the owners interviewed reported health concerns related to feeding a RMBD (gastrointestinal signs were the most common), even if the vast majority (94%) had previously declared RMBDs safe. The failure to recognize that the consumption of RMBDs may be a risk factor for the development of illness in pets was also reported by Connolly and colleagues [12], who found that 65% of the dog breeders interviewed disagreed with such statement. Owners should be counseled on the safety risks resulting from feeding RMBDs because many cases of pet foodborne disease and other health issues have been reported in literature [3]. Also, very few respondents said they would avoid administering RMBDs to puppies, ill dogs, and pregnant or lactating bitches. Nutritional imbalances are very common in this type of diet [21, 22] and the effects of prolonged administration could be more detrimental in young or sick dogs; furthermore, similarly to as in humans, dogs with impaired immune systems could show a decreased resistance to pathogens that leads to the development of foodborne illnesses.

The biggest advantage of RMBDs (57% of the answers) appeared to be the total control the owner has over the pet’s diet obtained by procuring its ingredients autonomously. Surprisingly, very few people named highly popular RMBD features like palatability, absence of grains, or rawness of the ingredients as a main advantage. The reasons why owners feeding RMBDs favor raw over cooked home-prepared diets should be better investigated, and studies comparing the benefits of one versus another would help understand whether such preference is correctly motivated. It appeared that even if owners grant highest priority to their management of the RMBD, ingredient collection and preparation time were indicated as the main drawback rather than health risks to the dog.

The present study revealed that only 14% of the persons interviewed had asked a veterinarian or a nutrition-trained expert for advice on formulating a RMBD. Most owners created their own recipes following other peoples’ advice or guidelines available online or in books and magazines. This should be discouraged, because some studies [23–25] have shown such published recipes of home-prepared diets for dogs and cats to have multiple nutritional imbalances (i.e. 95 to 100% of the recipes analyzed failed to meet all essential nutritional requirements for the target animals). More specifically, two other studies demonstrated most self-formulated home-prepared RMBDs to be nutritionally inadequate, and multiple mineral and vitamin imbalances (especially for calcium, phosphorous and vitamin D) were shown in respectively 100 and 76% of the recipes considered [21, 22]. Furthermore, a minority of the owners we surveyed (1%) said they followed the feeding instructions provided on the labels of the commercial RMBDs they purchased. It must be noted however that even if the labels claim they meet the requirements for growth, reproduction, and maintenance [21], commercially available RMBDs also have significant mineral and vitamin imbalances. The long-term consumption of nutritionally incomplete and unbalanced diets could result in severe concerns to canine health, especially in young individuals. Taylor et al. [26] reported vitamin D–dependent rickets and suspected nutritional secondary hyperparathyroidism in an 8-month-old Shetland Sheepdog consuming an incomplete and unbalanced home-prepared RMBD. Moreover, the fact that only 21 owners declared using a mineral and vitamin supplement might suggest that most RMBDs prepared by the participants were incomplete and unbalanced. As already argued by Connolly and colleagues [12], the low frequency with which pet owners and breeders consult board-certified nutritionists is quite concerning. Other health issues associated with the composition of RMBDs have been reported along with nutritional deficiencies. Köhler and colleagues [27] described 12 dogs showing elevated serum thyroxine values, six of which developed clinical signs of hyperthyroidism due to the inclusion of raw beef gullet with thyroid tissue; thyroxine concentrations returned within the reference range in all dogs after changing the diet [27]. Again, many cases of fractured teeth and gastrointestinal obstructions or perforations were reported following the consumption of RMBDs containing bones [18–20], and this survey showed that 92% of the dogs received bones regularly.

It is noteworthy that 28% of the participating owners ordered RMBD products via internet. Online purchase of pet food has become very popular in recent years, and particular care should be taken with fresh raw products because they should be handled with scrupulous hygiene criteria and require the cold chain to remain uninterrupted. Moreover, the risk of microbiological growth increases due to the probability of higher temperatures during transport [28]. Regarding ingredients, beef and poultry were the meats most commonly included in RMBDs, and offal were found to comprise an important part, as 86% of the owners used them regularly, especially liver and heart. Other common ingredients were green tripe, probably due to its attributed probiotic property, and animal origin protein sources like fish, egg, and yoghurt. Fruits and vegetables were shown to play a marginal role, and carbohydrate sources were not very common despite a mild preference for potatoes. Interestingly, garlic was seen to be popularly used as a supplement despite the fact that evidence of its toxicity has been documented [29].

The present study had certain limitations. Although the online survey was an efficient approach to directly target pet owners feeding RMBDs, electronic recruitment through a social media site may have led to an under-representation of people who did not use such resources. Additional studies are needed to determine whether the feeding practices and motivations investigated in this study are representative of all RMBD-feeding dog owners in Italy and other countries.

## Conclusions

Dog owners’ growing interest in RMBDs appears to be mainly motivated by the intention to feed their animals a more natural and healthier diet. The benefits derived from the administration of raw foods that owners commonly report must be verified by future studies. Whereas the search for a feeding regimen alternative to commercial pet food is defensible, choosing a diet that puts both animal and human health at risk should not be encouraged. As revealed by this study, most owners underestimate the risks posed by feeding RMBDs and often rely on questionable sources such as the internet to gather information on pet nutrition. Public health agencies need to become more aware of the popularity gained by RMBDs and emphasize the educational role of the veterinarian, whose consultation may help owners make better-informed decisions for their pets and themselves. Referral to a board-certified nutritionist for the formulation of complete RMBDs that avoid long-term nutritional imbalances should be promoted as well.

## Methods

### Participant recruitment and survey design

In order to recruit owners who feed their dogs RMBDs, a web-based survey in Italian language was created using an online survey tool (Google Forms). The online questionnaire was piloted among the authors before its launching on the web but the results of this test were not included in the analysis. The link to the open survey was shared through groups of RMBD-feeding dog owners on a social media website (Facebook) for 4 weeks between September and October 2015 and no individuals had access later in the questionnaire period. Although the survey was intended solely for people who feed RMBDs to their dogs, no other exclusion criteria were fixed. The respondents came from a convenience sample of owners who saw the online link and freely volunteered to participate in the study.

The questionnaire contained 44 closed questions (43 of which were mandatory, only one was optional) and was divided into three sections. In the first section, information on the owner (personal and household data) was collected; in the second, the dog’s signalment (breed, age, sex, body weight, health status) was entered; the third part of the survey queried dog owner attitudes regarding RMBDs. More specifically, the questions in the final section were intended to determine the motivations for the feeding of RMBDs, the effects on the dog perceived, the level of awareness of associated risks, the positive and negative aspects of handling a RMBD, and details on formulations and ingredients.

No approval by an institutional review board was required because enrollment was on a voluntary basis and the participants consented to anonymous information collection.

Survey participants were instructed that only one member of a household should complete the questionnaire and that every respondent could be responsible for just one dog.

All data were checked to verify that no multiple entries from the same individual were included. This was done by comparing respondents’ variables (such as time of submission, postcode area, gender, age etc.). The study was open to Italian-speaking owners, but no respondents were excluded because their postcode or hometown were outside Italy. Respondents were not allowed to submit incomplete questionnaires, which were automatically deleted without being recorded.

The translated survey is available as a supplementary material (Additional file 1).

### Data analysis

The data collected from the survey were transferred into a spreadsheet (Excel, Microsoft) and submitted to descriptive analysis.

## Additional file

Additional file 1: Translated Survey. This document provides the questionnaire translated into English. The questions were numbered sequentially, from number 1 to 44, and were divided into three sections (1–9: information about the owner; 10–17: dog’s signalment; 18–44: dog owner’s attitudes regarding RMBDs). If multiple answers were possible, this was specified in brackets after the question. (DOCX 19 kb)

## Abbreviations

RMBDs: Raw Meat-Based Diets
SD: Standard Deviation
