# Supplementary material for: Raw meat-based diets for dogs: survey of owners’ motivations, attitudes and practices
Source: BMC Vet Res. 2019 Mar 4;15:74. doi: 10.1186/s12917-019-1824-x (PMC6399943; doi:10.1186/s12917-019-1824-x)
Supplement: Supplementary file 1 — Translated Survey. This document provides the questionnaire translated into English. The questions were numbered sequentially, from number 1 to 44, and were divided into three sections (1–9: information about the owner; 10–17: dog’s signalment; 18–44: dog owner’s attitudes regarding RMBDs). If multiple answers were possible, this was specified in brackets after the question. (DOCX 19 kb) [file 12917_2019_1824_MOESM1_ESM.docx]

Survey

Owner data

1. District
   _____________________________
2. Sex
   - Male
   - Female
3. Age
   - <20 years old
   - 21-40
   - 40-60
   - >60
4. Household
   - Single
   - Couple
   - Family with children
   - Housemates with no kinship
   - Other ______________________________
5. At home there are currently
   - Children <3 years old
   - Children 3-10 years old
   - Elderly >75 years old
   - Pregnant women
   - Chronically ills or in therapy
6. You live in a house
   - With a garden
   - With a big terrace
   - With no outdoor space
7. To exercise daily, the dog
   - Stays in the garden/terrace
   - Stays in the fenced area
   - Is walked by someone
   - Other ______________________________
8. How did you come to know the raw meat-based diet? (multiple answers possible)
   - Suggested by friends
   - Doing some research on the internet
   - Suggested by the veterinarian
   - Reading magazines, books and articles
   - Suggested by the breeder
   - Other ______________________________

Dog signalment

1. Breed
   ______________________________
2. Body weight (kg)
   ______________________________
3. Sex
   - M
   - F
4. Sterilization
   - Yes
   - No
5. Age
   ______________________________
6. Size
   - Small (<10 kg)
   - Medium (11-25 kg)
   - Large (25-45 kg)
   - Giant (>45 kg)
7. Does the dog live indoor?
   - Yes, always
   - No, always outdoor (garden/kennel/fenced area)
   - Only at certain times of the day
8. According to you, the dog is
   - Very underweight
   - Slightly underweight
   - Ideal
   - Slightly overweight
   - Very overweight
9. How often do you take the dog to the Veterinarian?
   - Only to get him vaccinated
   - Just in case of health concerns
   - Rarely or never
   - To get him vaccinated and in case of health concerns
   - Other _______________________________

Raw meat-based diet (RMBD)

1. What is the main reason why you chose to provide a RMBD to your dog?
   - I do not trust commercial pet food
   - Commercial pet food caused health problems in the past
   - The dog does not eat commercial pet food
   - A matter of principle: to respect the carnivorous nature of the dog (dogs descend from wolves)
   - I heard that this kind of diet causes less health problems in dogs
   - Other ______________________________
2. From a sanitary point of view, do you think RMBDs are safe?
   - Yes
   - No
   - I never had the issue raised
3. According to you, which is the main advantage of a RMBD?
   - It contains mainly animal origin proteins, which are the most important component of dogs’ diet
   - It does not include carbohydrates
   - The main ingredients are raw
   - The dog likes it more than kibbles
   - The dog is more satisfied because it takes a longer time to be consumed
   - I have the total control over the diet and I am fully aware of its composition
   - Other ______________________________
4. According to you, which is the main disadvantage of a RMBD?
   - The risk of bone ingestion and the consequent health problems
   - The dog does not always appreciate it
   - Sometimes it causes diarrhea or vomiting in the dog
   - The recruitment of certain ingredients is complicated sometimes
   - It is expensive
   - It takes a long time for the preparation
   - Other ______________________________
5. Why don’t you feel good about commercial pet food? (multiple answers possible)
   - I don’t know which ingredients are included
   - I don’t know origin and quality of the raw materials included
   - It contains carbohydrates
   - The dog consumes the meal too fast and is not satisfied
   - It contains additives
   - The dog does not like it
   - I prefer giving my dog fresh ingredients
   - I like cooking for my dog
   - Other ______________________________
6. Is it true that dogs can get ill by eating raw meat?
   - Yes
   - No
   - Never heard about it
   - I heard about it but I am not concerned
7. How long have you been feeding RMBs?
   - Less than one year
   - 1-5 years
   - 5-10 years
   - More than 10 years
8. Have you ever feed commercial pet foods before choosing to feed RMBDs?
   - Yes
   - No
9. Where do you usually purchase raw meat? (multiple answers possible)
   - Supermarket
   - butcher shop
   - online shops
   - slaughterhouse
   - at any of these indistinctly
10. Which meat types do you usually purchase? (multiple answers possible)
    - Lamb
    - Pork
    - Beef
    - Chicken
    - Turkey
    - Horse
    - Game (duck, quail, etc.)
    - Rabbit
    - Other ________________________________
11. Which fish types do you usually purchase? (multiple answers possible)
    - Anchovies
    - Cod
    - Salmon
    - Sardines
    - Mackerel
    - Herrings
    - Other ________________________________
12. Which meat parts do you usually purchase? (multiple answers possible)
    - Minced meat
    - Green tripe
    - White tripe
    - Thigh
    - Shoulder
    - Loin
    - Neck (whole or minced)
    - Tail
    - Sirloin
    - Other ________________________________
13. Which offal do you usually purchase? (multiple answers possible)
    - Liver
    - Kidney
    - Heart
    - Lung
    - Spleen
    - Giblets
    - Other ________________________________
14. Do you feed vegetables? (state also the administration frequency: 0=never; 1= Rarely, one-three times/month; 2=once a week; 3=often, two-three times/week; 4=daily)
    - Zucchini 0 1 2 3 4
    - Green beans 0 1 2 3 4
    - Squash 0 1 2 3 4
    - Carrots 0 1 2 3 4
    - Beans 0 1 2 3 4
    - Leafy vegetables 0 1 2 3 4
    - Cabbage 0 1 2 3 4
    - Celery 0 1 2 3 4
    - Artichokes 0 1 2 3 4
    - Eggplant 0 1 2 3 4
    - Bell peppers 0 1 2 3 4
    - Peas 0 1 2 3 4
15. Do you feed fruit? (state also the administration frequency: 0=never; 1= Rarely, one-three times/month; 2=once a week; 3=often, two-three times/week; 4=daily)
    - Apple 0 1 2 3 4
    - Pear 0 1 2 3 4
    - Citrus 0 1 2 3 4
    - Banana 0 1 2 3 4
    - Melon 0 1 2 3 4
    - Watermelon 0 1 2 3 4
    - Peach 0 1 2 3 4
    - Apricot 0 1 2 3 4
    - Pineapple 0 1 2 3 4
    - Strawberry 0 1 2 3 4
    - Fig 0 1 2 3 4
16. Do you feed any carbohydrate source? (multiple answers possible)
    - Puffed rice
    - Potatoes
    - Bread
    - Pasta
    - Barley
    - Rice
    - Mixed cereals
    - No
    - Other ________________________________
17. Mark the supplements you use: (multiple answers possible)
    - Bone meal
    - Algae
    - Brewing yeast
    - Garlic
    - Salmon oil
    - Linseed oil
    - Cod liver oil
    - Coconut oil
    - Bone broth
    - Vitamin and mineral powder
    - Nuts
    - None
    - Other ________________________________
18. Do you feed your dog bones?
    - Yes, everyday
    - Yes, a few times a week
    - Rarely
    - No, I think it could be dangerous
    - Other ________________________________
19. Mark the other animal origin ingredients you use: (multiple answers possible)
    - None
    - Butter
    - Mascarpone
    - Yoghurt
    - Cheese
    - Milk
    - Egg
    - Tallow
    - Lard
    - Other ________________________________
20. Please specify the cheese types you use, if any
    - _____________________________________
21. Do you still feed some kibbles?
    - No, I completely eliminated kibbles
    - Yes, I still use some everyday
    - Regularly, about half of the daily ration
    - Regularly, more than half of the daily ration
    - Only when I travel for convenience
22. How do you calculate the amount of each ingredient included in the RMBD you feed?
    - I gauge by sight, I feel I’ve become skilled
    - I rely on nutritional guidelines published in websites or in books
    - I search for receipts online
    - I rely on the guidelines provided by the online sellers of the products I buy
    - I ask for my veterinarian’s advice
    - I learnt to formulate diets by myself, following other people’s advice available online
    - Other ________________________________
23. How much time do you spend preparing a RMBD?
    - Not much, I just defrost the ones I purchase
    - I am not able to quantify the time I spend
    - 10 minutes a day
    - 20-30 minutes a day
    - 30-45 minutes a day
    - 45-60 minutes a day
    - 60-120 minutes a day
    - >120 minutes a day
24. Did you notice any health improvement in your dog since you’ve been feeding a RMBD? (multiple answers possible)
    - Better-looking coat and skin
    - Cleaner teeth
    - Vitality and temper
    - Performance (training, competitions, focus,…)
    - Reproductive efficiency
    - Muscle mass
    - Odorless breath
    - Cleaner ears
    - Other ________________________________
25. Did you notice any health concern in your dog since you’ve been feeding a RMBD? (multiple answers possible)
    - Constipation
    - Vomiting
    - Abdominal pain
    - Diarrhea
    - Allergies
    - Hair loss
    - Oral lesions
    - Teeth damage
    - Bone splinters
    - Other ________________________________
26. Did you notice any behavioral change in your dog since you’ve been feeding a RMBD? (multiple answers possible)
    - Never
    - Calmer
    - Livelier and more active
    - More nervous
    - More aggressive
    - Other ________________________________
27. If you own more dogs, do you avoid feeding a RMBD in certain circumstances? (multiple answers possible)
    - I feed everyone a RMBD, indistinctly
    - I avoid feeding puppies a RMBD
    - I avoid feeding senior dogs a RMBD
    - I avoid feeding pregnant bitches a RMBD
    - I avoid feeding lactating bitches a RMBD
    - I avoid feeding ill dogs a RMBD
    - Other ________________________________
